# Granulomatous gall bladder: A surgico-pathological challenge

**DOI:** 10.4103/0256-4947.62831

**Published:** 2010

**Authors:** Roobina Khan, Shaista M Vasenwala, Shaukat H. Arif, Syed H. Harris

**Affiliations:** From the Departments of Pathology and Surgery, Jawaharlal Nehru Medical College, Aligarh Muslim University, Aligarh 202002, Utter Pradesh, India

**To the Editor:** *Mycobacterium tuberculosis* can affect any part of the gastrointestinal tract from the mouth to the anal canal along with the other organs of the peritoneal cavity, but the gall bladder is unlikely to be primarily involved. The incidence of localization of the tubercular bacilli in the gall bladder is uncommon as the intact gall bladder mucosa is resistant to the bacterium because of the concentrated bile acids present in the organ. The spread of infection to the gall bladder could be hematogenous, from an adjacent caseating lymph node or from the peritoneum.^1^ Cholilithiasis or cystic duct obstructions are thought to be essential for the development of gall bladder tuberculosis.^1^,^2^

**Figure 1:**
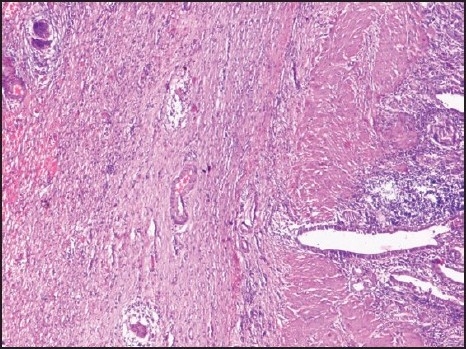
Section from the gall bladder showing multiple epithelioid granulomas and Langhans giant cells in the serosa with lymphocytic infiltration in all the layers (hematoxylin and eosin, ×50).

A 26-year-old female presented to the surgical outpatient department with right upper on-and-off abdominal and epigastric pain with dyspepsia for 8 months. After taking a detailed clinical history, ultrasonography of the abdomen revealed a thickened gall bladder wall with multiple gall stones, leading to a diagnosis of chronic cholecystitis with cholelithiasis. Gastroduodenoscopy of the patient was normal. X-ray of the chest and all other hematological parameters were also normal. The patient underwent a routine laparoscopic cholecytectomy, which was uneventful. After the extraction of the gall bladder, the specimen was opened to reveal a palpable nodular induration in the wall at the fundus of the organ. As facilities for frozen section biopsy were unavailable, and the possibility of malignancy was present, an imprint cytology was sent, which demonstrated abundant epithelioid-like cells with some necrosis. No malignant cells were seen. The surgery was terminated and the histopathology closely followed. Tuberculosis of the gall bladder showing caseous necrosis with Langhans' giant cells with epithioloid cells and lymphocytes (Figure 1) was diagnosed. Polymerase chain reaction (PCR) for tuberculosis confirmed the diagnosis. The patient was put on standard antitubercular chemotherapy for 9 months, after which she had regular follow-ups and was asymptomatic at 11 months after surgery.

The first case of tuberculosis of the gall bladder was described in 1870 by Gaucher.^3^ Gastrointestinal tuberculosis usually presents as involvement of the peritoneum, intestine, or the lymph nodes, and isolated tuberculosis of the gall bladder is extremely rare with only about 50 cases reported in literature.^4^ In a study of 280 patients with hepatobiliary tuberculosis, only one case had gall bladder involvement.^5^ In their experience over a period of two decades with gall bladder and pancreatic tuberculosis, Saluja et al^6^ found only three cases of the gall bladder being involved with the tuberculous infection. The rarity of the disease involving the gall bladder could be attributed to the fact that bile is thought to have an inhibitory effect on the gall bladder mucosa for the development of tuberculosis. Cholelithiasis and cystic duct obstruction are considered important factors for its development,^1^,^2^ as seen in our patient. There are very few reports in literature of acalculous cholecystitis with tuberculosis.^7^

There are no pathognomic features for the preoperative diagnosis of gall bladder tuberculosis^3^ and accidental reporting on histopathology has been a common occurrence.^2^ This case is reported to emphasize the opening of the gall bladder in the operating room after its extraction from the peritoneal cavity because of the suspicion of any intraluminal lesions, and thus to alert the reader of this rare pathology.
